# Two Distinct Pathways in Mice Generate Antinuclear Antigen-Reactive B Cell Repertoires

**DOI:** 10.3389/fimmu.2018.00016

**Published:** 2018-01-22

**Authors:** Martin Faderl, Fabian Klein, Oliver F. Wirz, Stefan Heiler, Llucia Albertí-Servera, Corinne Engdahl, Jan Andersson, Antonius Rolink

**Affiliations:** ^1^Developmental and Molecular Immunology, Department of Biomedicine, University of Basel, Basel, Switzerland

**Keywords:** antinuclear antibodies, autoantibodies, monoclonal antibodies, mouse model, systemic lupus erythematosus-like disease, somatic hypermutation

## Abstract

The escape of anti-self B cells from tolerance mechanisms like clonal deletion, receptor editing, and anergy results in the production of autoantibodies, which is a hallmark of many autoimmune disorders. In this study, we demonstrate that both germline sequences and somatic mutations contribute to autospecificity of B cell clones. For this issue, we investigated the development of antinuclear autoantibodies (ANAs) and their repertoire in two different mouse models. First, in aging mice that were shown to gain several autoimmune features over time including ANAs. Second, in mice undergoing a chronic graft-versus-host disease (GVHD), thereby developing systemic lupus erythematosus-like symptoms. Detailed repertoire analysis revealed that somatic hypermutations (SHM) were present in all Vh and practically all Vl regions of ANAs generated in these two models. The ANA B cell repertoire in aging mice was restricted, dominated by clonally related Vh1-26/Vk4-74 antibodies. In the collection of GVHD-derived ANAs, the repertoire was less restricted, but the usage of the Vh1-26/Vk4-74 combination was still apparent. Germline conversion showed that the SHM in the 4-74 light chain are deterministic for autoreactivity. Detailed analysis revealed that antinuclear reactivity of these antibodies could be induced by a single amino acid substitution in the CDR1 of the Vk4-74. In both aging B6 and young GVHD mice, conversion of the somatic mutations in the Vh and Vl regions of non Vh1-26/Vk4-74 using antibodies showed that B cells with a germline-encoded V gene could also contribute to the ANA-reactive B cell repertoire. These findings indicate that two distinct pathways generate ANA-producing B cells in both model systems. In one pathway, they are generated by Vh1-26/Vk4-74 expressing B cells in the course of immune responses to an antigen that is neither a nuclear antigen nor any other self-antigen. In the other pathway, ANA-producing B cells are derived from progenitors in the bone marrow that express B cell receptors (BCRs), which bind to nuclear antigens and that escape tolerance induction, possibly as a result of crosslinking of their BCRs by multivalent determinants of nuclear antigens.

## Introduction

A hallmark of the autoimmune disease systemic lupus erythematosus (SLE) is the presence of antinuclear autoantibodies (ANAs) in the serum (1–6). These antibodies are directed against histones, DNA, histone–DNA complexes, and various ribonuclear complexes (anti-SM, anti-Ro, and anti-La) (3–6) and may be found in immune complexes that play an important role in the pathogenesis of SLE. The disease occurs more frequently in females than males (ratio 10:1) with a peak incidence at 45–65 years. Based on the findings that ANA producing B cells have undergone Ig class switching and carry large numbers of somatic mutations, it is very likely that ANAs arise from B cells participating in T cell dependent antigen responses (3–6).

Studies using mouse models spontaneously developing an SLE-like disease have improved our knowledge of the etiology of this disease (7–9). In particular, these studies have highlighted the complex genetic contribution to the development of the disease as well as the important role of somatic mutations of antibody genes in the formation of autoantibodies (7–13).

The generation of a self-tolerant B cell repertoire is critically dependent upon the processes of clonal deletion, receptor editing, and anergy (14–19). Exactly how B cells escape central tolerance is, however, still not completely understood. Ample evidence has been provided indicating that non-autoreactive B cells can become autoreactive through somatic mutations in their variable heavy (Vh) and light (Vl) chain regions (6, 10, 13, 20). Equally, B cells using germline-encoded Vh and Vl regions escaping central tolerance induction in the bone marrow could also generate autoreactive B cells (21).

Recently we showed that almost all aging (8–12 months old) C57BL/6 (B6) mice develop several features characteristic of autoimmunity. This included germinal center formations in the spleen, kidney depositions of IgM, lymphocyte infiltrates in the salivary glands, as well as the production of high titers of IgG ANAs. Furthermore, this IgG ANA generation was shown to be T cell dependent (22). However, aging B6 mice do not develop real signs of disease. Here, we compare the ANA B cell repertoire of such aging B6 mice with that of (B6 × B6.*H-2^bm12^*)F_1_ mice undergoing a chronic graft-versus-host disease (cGVHD) and thus developing an SLE-like disease and death (23). Results indicate that the ANA B cell repertoire of aging B6 mice is more restricted than that of mice undergoing GVHD and is only partially overlapping. Moreover, we show that the ANA producing B cells in aging mice and in GVHD mice are derived from progenitors expressing B cell receptors (BCRs) either recognizing or not recognizing nuclear antigens. These findings indicate that ANA producing B cells in both aging mice and in GVHD mice are generated by two pathways: by defective tolerance induction in the bone marrow or by hypermutation in the V-regions of B cells responding to a foreign antigen.

## Materials and Methods

### Mice and Induction of cGVHD

C57BL/6 and (C57BL/6 × B6(C)-*H2-Ab^bm12^*/KhEgJ)F_1_ mice were bred under specific pathogen free conditions in our animal unit. A cGVHD was induced by i.v. injection of 8 × 10^7^ spleen plus lymph node cells from B6 mice into 8–10 weeks old B6 × bm12 mice, following established protocols (23).

### Generation of Hybridomas

Spleen cells derived from aging B6 mice (aged 8–12 months) or B6 × bm12 mice undergoing a cGVHD were fused to the Sp2/0-Ag14 fusion partner following standard protocols. In brief, 2 × 10^7^ Sp2/0 cells were used for the PEG1500 (Roche Diagnostics)-mediated fusion of all lymphoid cells prepared from an entire spleen. The fused cells were plated into 25 flat-bottom 96-well plates containing 200 µl HAT-medium (2%FBS; GIBCO, 2% IL6, in house, 1× HAT supplement, Sigma) per well and incubated at 37°C in 10% CO_2_ in air. After 10–12 days, supernatants were tested for IgG production by ELISA and for antinuclear reactivity by immunofluorescence (see below). Cells from IgG ANA positive wells were thereafter sub-cloned at limiting dilution. We routinely obtained fusion frequencies between 10^−3^ and 5 × 10^−3^. Thus, for each mouse, we have screened between 5,000 and 20,000 hybrids for IgG ANA production. Since the vast majority of B cell hybrids produce IgM, we tested for the success of sub-cloning by performing simultaneous ELISA for IgM and IgG (see below) in supernatants of growing clones. The IgG containing supernatants were re-tested for ANA reactivity, before being further processed for Ig V-gene analyses (see below).

### Determination of IgG Sub-Class and Anti-Histone/DNA/Sm/SS-B/La Reactivity

Determination of IgG sub-class, l-chain, and detection of anti-DNA antibodies was done by standard ELISA. For determination of anti-histone, anti-Sm or anti-SS-B/La ELISA plates were coated with 2.5 μg/ml of the respective antigens in PBS (all purchased from Immunovision). Alkaline phosphatase labeled goat anti-mouse IgG, goat anti-mouse l-chain or goat anti-human IgG (Southern Biotech) was used for detection. The ELISA was performed as previously described (24–26).

### Antinuclear Autoantibody Determination

Kidney cryosections from Rag2^−/−^ mice of homozygous matings were incubated with supernatants or purified antibodies as described in Ref. (27). For detection, either FITC labeled goat anti-mouse IgG (Jackson ImmunoResearch) or FITC labeled rabbit anti-human IgG (Jackson ImmunoResearch) were used.

### Vh and Vl Sequencing Analysis

RNA from ANA positive hybridomas was extracted using TRI Reagent (Sigma) followed by cDNA synthesis (GoScript Reverse Transcriptase) according to the manufacturer’s protocol and using primers as specified in Table S5 in Supplementary Material. Amplification of the heavy and light chain V-regions was performed using Vent polymerase (New England Biolabs) and, subsequently, the Vh and Vl regions were ligated into the pJet1.2 blunt end cloning vector (Thermo Fisher Scientific). For sequencing, plasmids were sent to Microsynth (Balgach, Switzerland). Resulting sequences were inspected using DNASTAR and aligned to the germline heavy and light chain sequences of the international ImMunoGeneTics information system^®^ (http://imgt.org).

### Reversion of Somatic Mutations Back into Germline Configuration

Double-stranded DNA encoding the variable regions of heavy and light chains obtained from ANA positive hybridomas as well as their germline and mutated versions were ordered as gBlock gene fragments from IDT (Integrated DNA Technologies). These fragments were cloned into heavy and light chain expression vectors driven by the human cytomegalovirus promoter and containing the human IgG_1_ constant region for the heavy chain and the human kappa constant region for the light chain (28) (a kind gift from Dr. Hedda Wardemann, Deutsches Krebsforschungszentrum, Heidelberg, Germany). Subsequently, antibodies were produced in HEK 293 cells (ATCC, No. CRL-1573) and purified by affinity chromatography on Protein A Sepharose^®^ (GE Health Care, Uppsala, Sweden) as described (28, 29).

## Results

### IgG ANA Producing Hybridomas from Aging B6 Mice

Hybridomas were generated independently from spleen cells of seven individual aging B6 mice, which had high titers of serum ANA. The resulting IgG producing hybridomas were then tested for ANA reactivity by immunofluorescence, and positive cultures were sub-cloned. In total, 36 hybridomas producing IgG ANA were generated (Table 1). Of these, 16 were IgG_2a_, 19 were IgG_2b_, and only one was IgG_1_. This heavy chain selection suggests that IgG ANA formation in aging B6 mice is mainly driven by a T_h_1 response (30). All ANAs contained a kappa light chain. Sequence analysis of the corresponding Vh and Vk regions used by these hybridomas revealed a restricted repertoire (V, D, and J annotations are according to the IMGT data base). Thus, 25 (69.4%) hybridomas used the Vh1-26 gene and this usage was found among hybridomas of all seven individual mice. Moreover, a very high frequency of Vk4-74 gene usage was also found. Thus, 23 (63.9%) hybridomas, derived from 5 of 7 individual mice, used this particular Vk light chain gene. Strikingly, 21 of the 23 Vk4-74 expressing hybridomas expressed the Vh1-26 heavy chain. Thus, the IgG ANA B cell repertoire of aging B6 mice is dominated by those expressing a Vh1-26 heavy chain gene in combination with a Vk4-74 light chain gene.

**Table 1:** Characteristics of ANA-reactive mAbs derived from aging B6 mice.^a^^,^^b^

Mouse number	Hybridoma number	IgG isotype	Ig-heavy chain v-region			Ig-light chain v-region	
Aging B6 # 1	5.D3.D10	IgG2b	Vh1-26	D1-1	J4	Vk4-74	Jk4
	5.D3. E12	IgG2b	Vh1-26	D1-1	J4	Vk4-74	Jk4
	7.E8.B11	IgG2b	Vh1-26	D1-1	J4	Vk4-74	Jk4
	7.F9.F1	IgG2b	Vh1-75	D2-4	J2	Vk?	
	8G3.E6	IgG2a	Vh1-26	D1-1	J4	Vk4-74	Jk4
	8.H7.D3	IgG2b	Vh1-26	D1-1	J4	Vk4-74	Jk4
	15.G10.F10	IgG2b	Vh1-26	D1-1	J4	Vk4-74	Jk4
	15.H12.E4	IgG2b	Vh1-26	D1-1	J4	Vk4-74	Jk4
	16.B9.C9	IgG2b	Vh1-26	D1-1	J4	Vk4-74	Jk4
	16.D7.A9	IgG2a	Vh1-26	D1-1	J4	Vk4-74	Jk4
	17.A9.E6	IgG2b	Vh1-26	D1-1	J4	Vk4-74	Jk4
	22.C9.G7	IgG2b	Vh1-26	D1-1	J4	Vk4-74	Jk4

Aging B6 # 2	1.G5.B8	IgG1	Vh1-39	D4-1	J1	Vk4-57	Jk5
	8.G9.G4	IgG2b	Vh1-26	D1-1	J4	Vk6-32	Jk2

Aging B6 # 3	13.4.5.A	IgG2a	Vh1-26	D2-4	J3	Vk4-74	Jk2
	13.18.A	IgG2a	Vh1-26	D2-4	J3	Vk4-74	Jk2
	13.27.B	IgG2a	Vh1-26	D2-4	J3	Vk4-74	Jk2
	13.31A	IgG2a	Vh1-26	D2-4	J3	Vk4-74	Jk2
	13.69.B	IgG2a	Vh1-26	D2-4	J3	Vk4-74	Jk2
	13.85B	IgG2a	Vh1-26	D2-4	J3	Vk4-74	Jk2

Aging B6 # 4	1A2.1	IgG2a	Vh1-26	D2-2	J4	Vk4-74	Jk2
	2F8.1	IgG2b	Vh1-26	D1-1	J4	Vk4-74	Jk2

Aging B6 # 5	12.G3.G7	IgG2a	Vh1-26	D1-1	J4	Vk5-43	Jk2
	15.D10B3	IgG2a	Vh1-26	D1-1	J4	Vk5-43	Jk2

Aging B6 # 6	3.2.2A	IgG2b	Vh1-74	D2-4	J3	Vk6-23	Jk5
	3.10.1A	IgG2b	Vh1-26	D1-1	J2	Vk4-61	Jk1
	5.13.1A	IgG2b	Vh1-26	D1-1	J2	Vk4-74	Jk2
	6.15.1A	IgG2a	Vh8-12	D3-1	J1	Vk4-91	Jk2
	7.7.3A	IgG2b	Vh1-22	D2-2	J1	Vk10-96	Jk2
	20.15.1A	IgG2a	Vh1-50	D4-1	J2	Vk4-74	Jk2
	23.6.1A	IgG2a	Vh1-22	D2-2	J1	Vk10-96	Jk2
	24.18.1A	IgG2b	Vh1-22	D2-2	J1	Vk10-96	Jk2
	25.8.1A	IgG2b	Vh3-6	D3-3	J3	Vk4-74	Jk2

Aging B6 # 7	3.25A	IgG2b	Vh1-50	D2-4	J2	Vk?	
	3.36A	IgG2a	Vh1-80	D2-4	J3	Vk4-58	Jk2
	3.73A	IgG2a	Vh1-26	D1-1	J4	Vk4-74	Jk2

*^a^All the mAbs converted into germline sequences are boxed in orange*.

*^b^All the mAbs using Vh1-26 have been boxed in yellow and those using Vk4-74 in red*.

**Figure 1:**
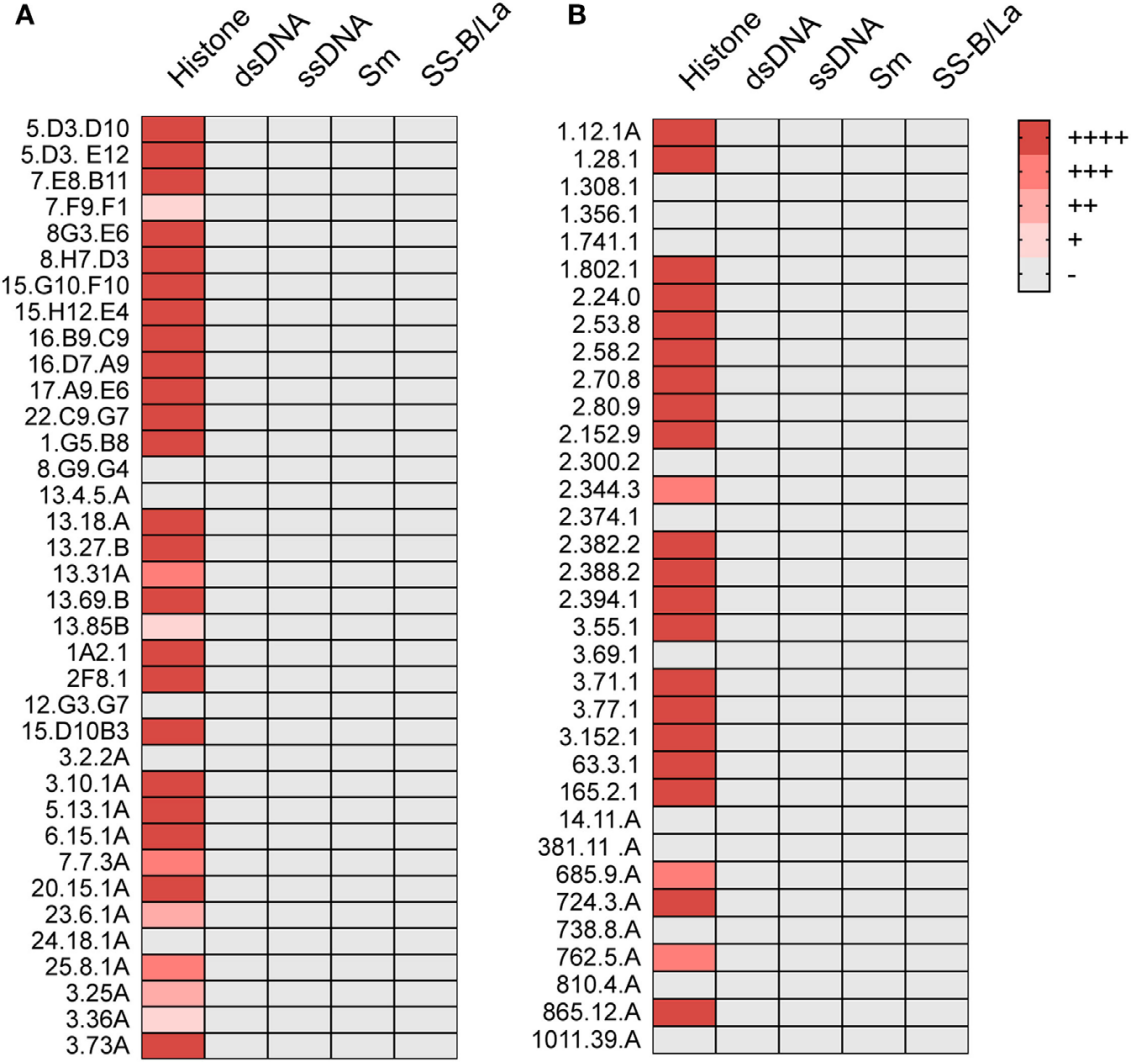
ANA reactivity is directed against histones. Histone, dsDNA, ssDNA, Sm, or SS-B/La binding of ANA-reactive mAbs derived from **(A)** aging B6 (Table 1) or **(B)** young B6 × bm12 mice undergoing a cGVH reaction (Table 2). Significant binding was defined as an O.D. of five times over background in ELISA. (−), no significant binding at 5 μg/ml; (+), binding at 5 μg/ml; (++), binding at 1.7 µg/ml; (+++), binding at 0.55 µg/ml; and (++++), binding at 0.19 µg/ml or lower.

We also tested these ANAs for their capacity to bind to histones, Sm antigens, SS-B/La antigens, and DNA. By ELISA, 26 of the 36 showed strong and 5 showed weak histone binding, whereas binding to the other nuclear antigens was undetectable (Figure 1A).

### In Individual Mice, Hybridomas Using Vh1-26/Vk4-74 Were Clonally Related

The fact that we obtained 11 hybridomas from mouse 1, and 6 hybridomas from mouse 3 using the Vh1-26/Vk4-74 combination with the same D, Jh, and Jk elements already strongly suggested a clonal relationship among the B cells that had fused to generate these hybridomas (Table 1). The finding that the amino acid sequences of the IgH and IgL CDR3 regions of these hybridomas were practically identical (Tables S1 and S2 in Supplementary Material) also supports this conclusion. Thus, at least in mouse 1 and 3, the ANA production seems to be dominated by a single B cell clone.

### IgG ANA from B6 × bm12 Mice Undergoing a Chronic Graft-versus-Host Reaction

We then asked whether the restricted IgG ANA B cell repertoire in aging B6 mice, which do not show obvious signs of disease, was similarly restricted in B6 × bm12 mice undergoing a chronic graft-versus-host reaction and, thus, developing an SLE-like disease. Hybridomas were independently generated from spleen cells of five mice with the highest ANA titers. These fusions resulted in 34 hybridomas with IgG ANA activity (Table 2). A total of 23 ANAs (68%) reacted to histones but again, none reacted to the other nuclear antigens (Figure 1B). IgG constant region usage analysis revealed a distribution rather similar to that in aged mice with 18 IgG_2a_, 13 IgG_2b_, and only 3 IgG_1_, the latter all from mouse 5. Thus, as in aging B6 mice, in B6 × bm12 mice undergoing a cGVHD, the IgG ANA formation appears to be Th1 cell driven. Again, all ANAs contained a kappa light chain.

**Table 2:** Characteristics of ANA-reactive mAbs derived from graft-versus-host disease (GVHD) mice.^a^^,^^b^

Mouse number	Hybridoma number	IgG isotype	Ig-heavy chain v-region			Ig-light chain v-region	
GVHD # 1	1.12.1A	IgG2b	Vh8-8	D2-14	J4	Vk4-74	Jk2
	1.28.1	IgG2a	Vh1-26	D1-1	J1	Vk4-74	Jk2
	1.308.1	IgG2a	Vh1-55	D2-4	J3	Vk3-4	Jk1
	1.356.1	IgG2a	Vh1-55	D2-4	J3	Vk3-4	Jk1
	1.741.1	IgG2a	Vh1-55	D2-4	J3	Vk3-4	Jk1
	1.802.1	IgG2b	Vh1-26	D1-1	J1	Vk4-63	Jk5

GVHD # 2	2.24.0	IgG2a	Vh1-52	D1-1	J1	Vk14-111	Jk5
	2.53.8	IgG2b	Vh14-2	D2-2	J2	Vk14-111	Jk1
	2.58.2	IgG2b	Vh14-2	D2-2	J2	Vk14-111	Jk1
	2.70.8	IgG2a	Vh14-4	D1-1	J3	Vk3-10	Jk1
	2.80.9	IgG2a	Vh14-4	D1-1	J3	Vk3-12	Jk1
	2.152.9	IgG2a	Vh1-52	D1-1	J1	Vk3-10	Jk1
	2.300.2	IgG2b	Vh1-55	D2-1	J2	Vk3-10	Jk5
	2.344.3	IgG2a	Vh1-52	D1-1	J1	Vk14-111	Jk5
	2.374.1	IgG2a	Vh1-26	D1-1	J1	Vk3-7	Jk1
	2.382.2	IgG2a	Vh14-4	D2-1	J3	Vk3-10	Jk1
	2.388.2	IgG2a	Vh1-31	D2-4	J4	Vk3-10	Jk2
	2.394.1	IgG2a	Vh14-4	D1-1	J3	Vk3-10	Jk1

GVHD # 3	3.55.1	IgG2a	Vh1-26	D1-1	J3	Vk4-74	Jk2
	3.69.1	IgG2b	Vh1-26	D1-1	J3	Vk4-74	Jk2
	3.71.1	IgG2a	Vh1-26	D2-5	J1	Vk4-74	Jk2
	3.77.1	IgG2a	Vh1-26	D1-1	J3	Vk4-74	Jk2
	3.152.1	IgG2b	Vh1-26	D1-1	J3	Vk4-74	Jk2

GVHD # 4	63.3.1	IgG2b	Vh1-55	D2-4	J2	Vk4-74	Jk2
	165.2.1	IgG2a	Vh8-12	D2-2	J1	Vk15-103	Jk5

GVHD # 5	14.11.A	IgG2b	Vh1-50	D1-1	J2	Vk1-117	Jk2
	381.11.A	IgG2b	Vh1-54		J4	Vk14-111	Jk2
	685.9.A	IgG2b	Vh1-59	D2-5	J3	Vk17-127	Jk5
	724.3.A	IgG1	Vh1-26	D1-1	J3	Vk4-91	Jk4
	738.8.A	IgG1	Vh1-26	D1-1	J3	Vk3-7	Jk1
	762.5.A	IgG2a	Vh5-17	D1-2	J4	Vk?	
	810.4.A	IgG1	Vh1-52	D2-12	J2	Vk3-10	Jk1
	865.12.A	IgG2b	Vh1-72	D3-3	J2	Vk4-74	Jk5
	1011.39.A	IgG2b	Vh1-53	D1-1	J2	Vk17-127	Jk5

*^a^All the mAbs converted into germline sequences are boxed in orange*.

*^b^All the mAbs using Vh1-26 are boxed in yellow and those using Vk4-74 in red*.

In aging B6 mice, a dominance of Vh1-26 and Vk4-74 usage by the IgG ANA producing hybridomas was observed. In B6 × bm12-derived hybridomas, the same genes were also found to be used, but at a much lower frequency. Thus, ten (29.4%) of these ANAs used Vh1-26 and nine (26.5%) used Vk4-74. Six of the hybridomas using Vk4-74 used the Vh1-26 heavy chain; however, five of these were derived from one mouse (GVHD mouse 3). These findings show that the IgG ANA B cell repertoires of aging B6 mice and B6 × bm12 mice undergoing a cGVHD are partially overlapping. However, the IgG ANA B cell repertoire seems to be more diverse in the B6 × bm12 mice than in the aging B6 mice. Therefore, the mechanisms underlying the generation of these autoreactive B cells might be different in the two model systems.

### Somatic Mutations in the Light Chain Determine the ANA Reactivity of Vh1-26/Vk4-74 Using mAbs

Sequence analysis revealed that most mAbs with ANA reactivity carried somatic mutations in their Vh and Vk regions. These results are summarized in Tables S1–S4 in Supplementary Material.

**Figure 2:**
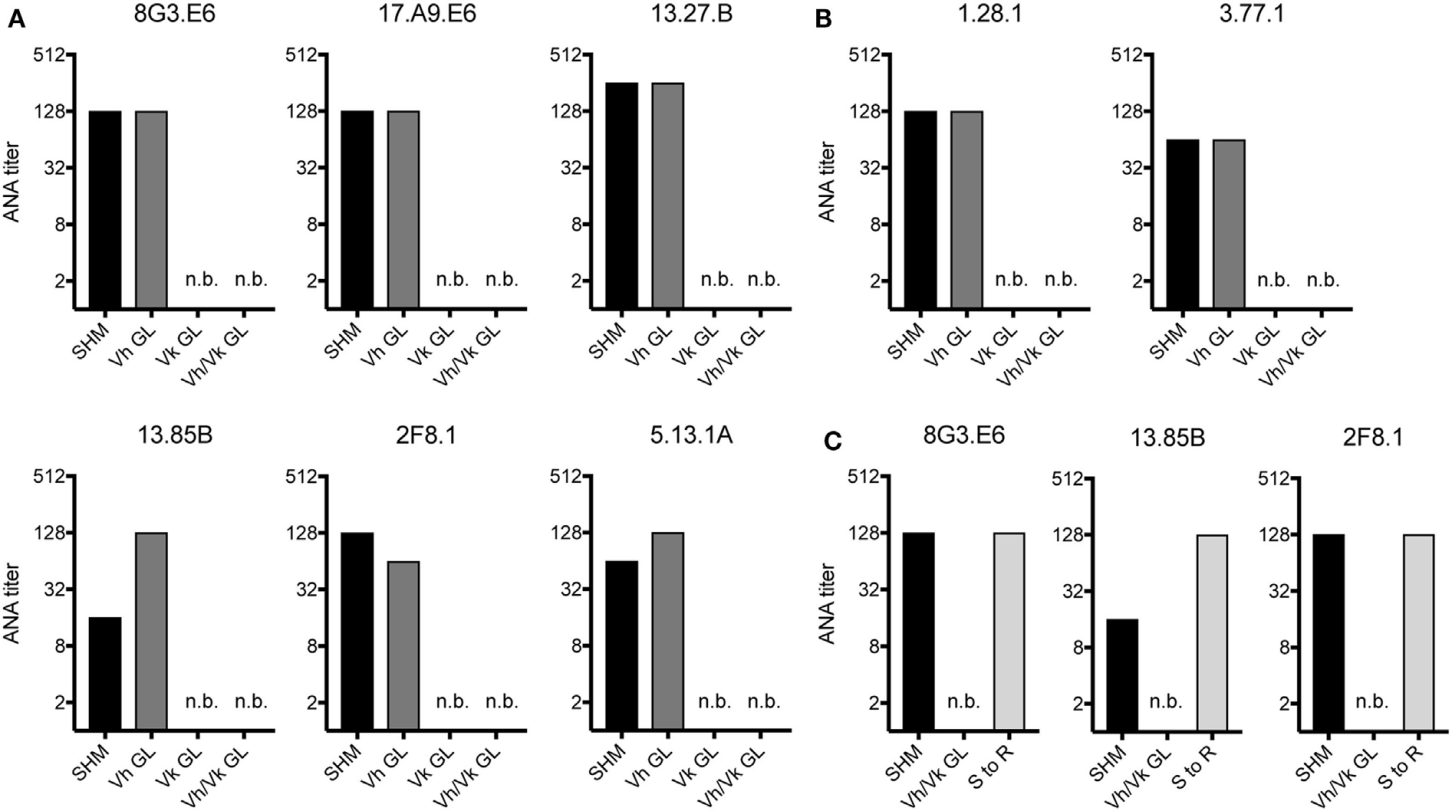
ANA reactivity of Vh1-26/Vk4-74 using mAbs is caused by mutations in the light chain. ANA titers of different Vh1-26/Vk4-74 mAbs derived from **(A)** aging B6 (Table 1) or **(B)** young B6 × bm12 mice undergoing a cGvH reaction (Table 2). The histograms depict titers of individual mAbs, either with the original SHM, or Vh and/or Vk in germline configuration (GL), or **(C)** having a single mutation in their Vk CDR1 resulting in a serine to arginine conversion. A titer of one was defined as binding at a concentration of 5 µg/ml mAb, followed by 1:2 serial dilution steps and recording the last dilution where binding still was possible. SHM, somatic hypermutation; GL, germline; n.b., no binding detectable at 5 µg/ml of mAb.

Since in aging B6 mice the ANA reactivity was dominated by mAbs expressing a Vh1-26/Vk4-74 heavy and light chain combination, we tested if somatic mutations in the Vh and/or the Vk regions of these mAbs were required for their autoreactivity. Therefore, the Vh and Vk regions of six (derived from 4 individual mice) mAbs of aging B6 and two mAbs of GVHD mice using the Vh1-26/Vk4-74 combination were reverted to their germline configuration. After expression and purification, the ANA titers of these reverted mAbs were directly compared to their original, mutated forms. As shown in Figures 2A,B, the ANA titers of all mAbs, in which the Vh region had been reverted into germline configuration but the Vk regions were still somatically mutated, behaved like the original ANAs, continuing to recognize the histone antigens. However, all mAbs where the Vh was still somatically mutated but the Vk had been reverted to germline configuration lost ANA reactivity. Also, as expected, all mAbs in which both Vh and Vkappa regions had been reverted to germline configuration lost ANA reactivity. Thus, the ANA reactivity of the Vh1-26/Vk4-74 mAbs is due to somatic mutations in the Vk4-74 gene.

Due to the finding that somatic mutations within the Vk4-74 gene determined the ANA reactivity of Vh1-26/Vk4-74 mAbs and in order to identify a common motif that could account for this autoreactivity, we analyzed the sequence of these Vk4-74 genes in more detail. This analysis revealed that 20 of 23 Vh1-26/Vk4-74 mAbs derived from aging B6 mice had a mutation at position 30 (IMGT numbering) in their CDR1 region of the Vk4-74 gene. The germline-encoded serine in these mAbs was mutated into a positively charged arginine residue. Introduction of such a serine to arginine mutation in non-autoreactive germline versions of three different mAbs resulted in a complete gain of ANA reactivity for all of them (Figure 2C). Thus, antinuclear reactivity of these mAbs can be induced by a single base pair substitution changing the serine at position 30 in the CDR1 of the Vk4-74 light chain into an arginine.

### B Cells Expressing a Germline-Encoded Immunoglobulin Vh Gene with a Negatively Charged CDR2 Region Contribute to the ANA-Reactive B Cell Repertoire

**Figure 3:**
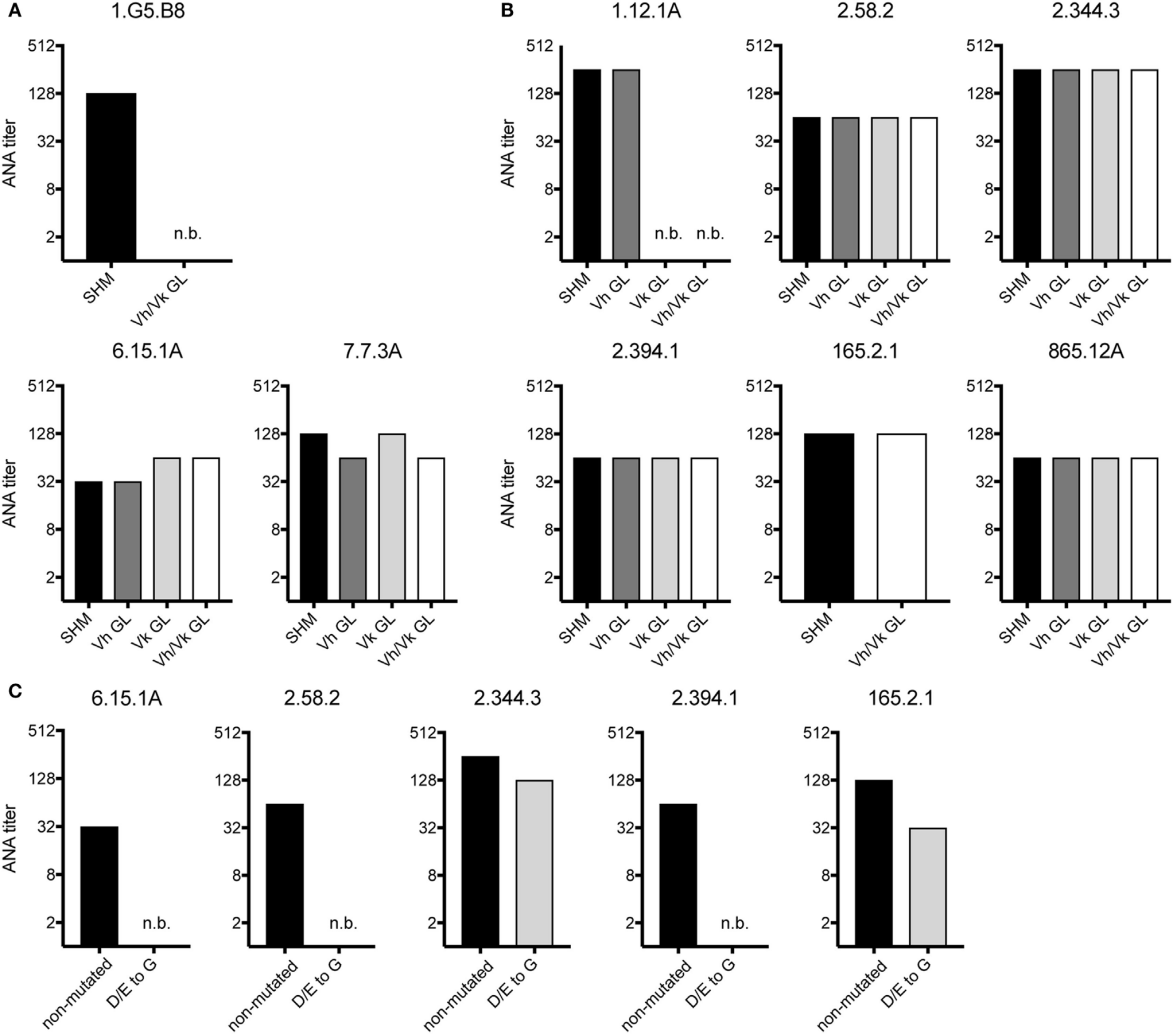
ANA reactivity of non-Vh1-26/Vk4-74 using mAbs is mainly germline encoded. ANA titers of different non-Vh1-26/Vk4-74 mAbs derived from **(A)** aging B6 or **(B)** young B6 × bm12 mice undergoing a chronic graft-versus-host disease. The histograms depict titers of individual mAbs listed in Table 3 containing V-regions, either with the original somatic hypermutations (SHM), or Vh and/or Vk in germline configuration (GL), or **(C)** containing a Vh CDR2 with conversions of aspartic and glutamic acids to glycine. Titers and legends as defined in Figure 2.

We also tested if non-Vh1-26/Vk4-74 using mAbs require somatic mutations in their Vh and/or Vk regions for ANA reactivity. Therefore, the Vh and Vk region of three mAbs of aging B6 mice and six mAbs of the GVHD mice were reverted to their germline configuration. The 1.G5.B8 mAb derived from an aging B6 mouse (No. 2) used a somatically mutated Vh region and a germline-encoded Vk region. Reversion of the Vh region of this mAb into germline sequences resulted in a complete abrogation of its ANA reactivity (Figure 3A). In contrast, upon reversion, the other two mAbs (7.7.3A and 6.15.1A, both from mouse 6) kept their ANA reactivity (Figure 3A). Thus, B cells expressing a germline-encoded immunoglobulin Vh can also contribute to the ANA-reactive B cell repertoire in aging B6 mice.

From the six GVHD-derived mAbs, one (1.12.1A, mouse 1) lost ANA reactivity upon reversion of the Vk but not the Vh gene into germline configuration (Figure 3B). The fact that this mAb uses the Vk4-74 gene indicates that this Vk gene can also give rise to ANA reactivity when paired with a Vh gene other than Vh1-26. The other five mAbs kept their ANA reactivity upon reversion of their Vh and Vk genes to germline configuration (Figure 3B). Thus, B cells expressing a germline-encoded immunoglobulin can also contribute to the ANA-reactive repertoire in mice undergoing a cGVHD.

A remarkable observation was that 5 of 7 mAbs (one from aging B6 mice and four from GVHD mice), which kept ANA reactivity upon complete reversion of their Ig genes into germline configuration, had a negatively charged Vh-CDR2 region as defined by having at least two more negatively than positively charged amino acids in this region (see Table 3). This observation prompted us to test if these negatively charged amino acids are involved in ANA reactivity. Therefore, the negatively charged amino acids in the CDR2 regions of these five mAbs were mutated into neutral glycine. As shown in Figure 3C, one mAb derived from an aging B6 mouse (6.15.1A) and two from a GVHD mouse (2.58.2 and 2.394.1), completely lost ANA reactivity upon aspartic acid/glutamic acid conversion into glycine. The ANA titers of the other two GVHD-derived mAbs (2.344.3 and 165.2.1) diminished by a factor of 2 and 3, respectively (Figure 3C). Thus, B cells expressing a germline-encoded immunoglobulin with a negatively charged Vh-CDR2 region contribute to the ANA-reactive repertoire. At this point, it is noteworthy that 6 of 11 (54.6%) of the non-Vh1-26 using ANA mAbs from aging B6 mice and 14 of 24 (58.3%) from mice undergoing cGVHD indeed carry a negatively charged Vh-CDR2 (Table 3).

**Table 3:** Vh-CDR2 regions of non-Vh1-26 ANA-reactive mAbs of graft-versus-host disease (GVHD) and aging B6 mice.^a^^,^^b^

Mouse number	Hybridoma number	Vh-region	Vh-CDR2 region	Mouse number	Hybridoma number	Vh-region	Vh-CDR2 region
GVHD # 1	1.12.1A	Vh8-8	I W W **D D D**	Aging B6 # 1	7.F9.F1	Vh1-75	I L P G S G S S
	1.308.1	Vh1-55	I Y P G S G S T				
	1.356.1	Vh1-55	I Y P G S G S T	Aging B6 # 2	1.G5.B8	Vh1-39	V N P N Y G T I
	1.741.1	Vh1-55	I Y P G S G S T				
				Aging B6 # 6	3.2.2A	Vh1-74	I H P S **D** S **D** T

GVHD # 2	2.24.0	Vh1-52	I **D** P S **D** G **E** T	Aging B6 # 6	6.15.1A	Vh8-12	I Y W **D D D K**
	2.53.8	Vh14-2	I **D** P **E D** G **E** T		7.7.3A	Vh1-22	I N P N N G G T
	2.58.2	Vh14-2	I **D** P **E D** G **E** S		20.15.1A	Vh1-50	I **D** P S **D** T F T
	2.70.8	Vh14-4	I **D** P **E** N G **D** T		23.6.1A	Vh1-22	I N P N N G **D** T
	2.80.9	Vh14-4	I **D** P **E** N G **D** T		24.18.1A	Vh1-22	I N P N N **D D** T
	2.152.9	Vh1-52	I **D** P S **D** G **E** T		25.8.1A	Vh3-6	I S C **D** G S S
	2.300.2	Vh1-55	I Y P G S V S T				
	2.344.3	Vh1-52	I **D** P S **D D E** T	Aging B6 # 7	3.25A	Vh1-50	I **D** P S **D** T Y T
	2.382.2	Vh14-4	I **D** P **E** N G **D** T	Aging B6 # 7	3.36A	Vh1-80	I Y P G **D** G **D** T
	2.388.2	Vh1-31	I F P Y N G V S				
	2.394.1	Vh14-4	I **D** P **E** N G **D** T				

GVHD # 4	63.3.1	Vh1-55	I Y P G S G S T				
	165.2.1	Vh8-12	I Y W **D D D K**				

GVHD # 5	14.11.A	Vh1-50	I **D** P S **D** S Y I				
	381.11.A	Vh1-54	I N P G S G G I				
	685.9.A	Vh1-59	I **D** P S **D** S S S				
	762.5.A	Vh5-17	I S **R** G S G I L				
	810.4.A	Vh1-52	I **D** P S **D** S **E** T				
	865.12.A	Vh1-72	I **D** P S S G G T				
	1011.39.A	Vh1-53	I N P S N **D** G T				
	1011.39.A	Vh1-53	I N P S N **D** G T				

*Bold font highlights negatively and positively charged amino acids*.

*^a^All mAbs that were converted into germline sequences or in which the negatively charged amino acids in the CDR2 were converted into glycine are marked with orange boxes*.

*^b^All the negatively charged amino acids in Vh-CDR2 region are marked in yellow and the positively charged amino acids in green*.

## Discussion

### Two Pathways to ANA-Producing B Cells

**Figure 4:**
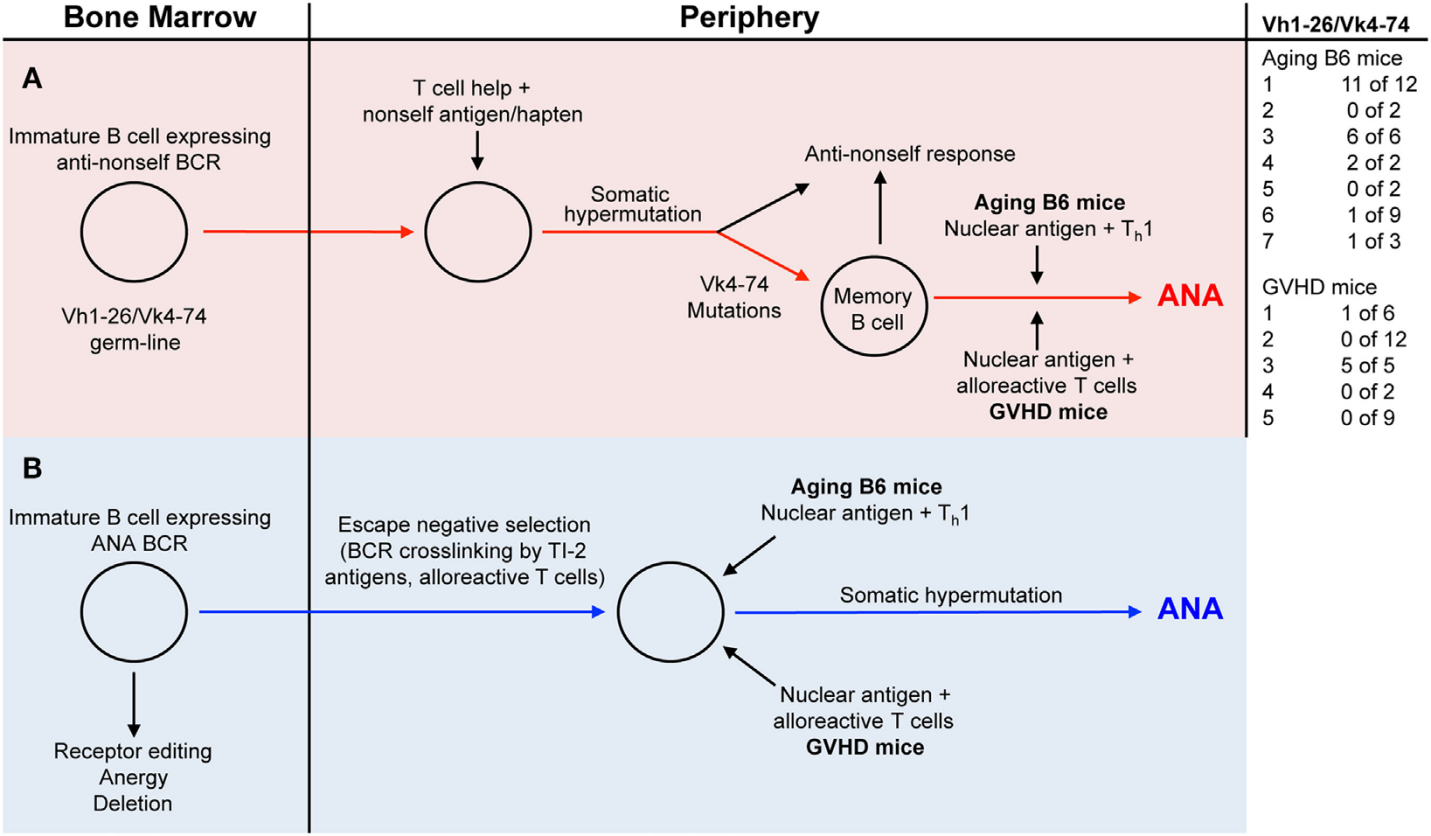
Two distinct pathways generate ANA producing B cells. [**(A)** red background] B cells with a B-cell receptor (BCR) using a germline-encoded Vh1-26 in combination with a germline-encoded Vk4-74 gets stimulated by a non-self (NS) antigen/hapten. Due to somatic hypermutations in the Vk4-74 gene, ANA-reactive memory B cells are generated. In aging B6 mice, these memory cells are then activated by nuclear antigens and Th1 cells to produce ANA. In the GVHD mice, nuclear antigens and alloreactive T cells activate these memory cells, resulting in ANA formation. [**(B)** blue background] Immature B cells in the bone marrow with germline-encoded BCRs specific for nuclear antigens escape tolerance induction mechanisms through BCR cross-linking by a T cell independent type 2-antigen (TI-2) antigens or a signal from alloreactive T cells. In the periphery of aging B6 mice, these cells get stimulated by nuclear antigens and Th1 cells to produce ANAs. In the periphery of GVHD mice, nuclear antigens and alloreactive T cells activate these cells to ANA formation.

In the present study, we compared antinuclear autoantibodies (ANAs) from aging B6 mice, which do not show signs of disease, with those derived from mice undergoing a chronic GVH reaction (GVHD mice) and which develop an SLE-like disease (23). In order to study the V-regions of ANAs, we generated B cell hybridomas from mice with high ANA titers in their serum. Almost all monoclonal ANAs bound to a mixture of histones. As expected from previous studies, ANA production was T cell and antigen dependent (1–3, 22), since virtually all V-regions of our monoclonal ANAs contained somatic mutations. To determine whether the ANA-producing B cells were derived from progenitors expressing B cell receptors (BCRs) for nuclear antigens, we reverted the mutated V-regions into the corresponding germline sequences and tested the resulting antibodies for ANA reactivity. Based on such analyses, we found that some ANA-producing B cells in both aging B6 mice and GVHD mice must be derived from progenitors expressing germline-encoded genes for BCRs specific for nuclear antigens, while several others were derived from progenitors expressing BCRs that showed no ANA reactivity. The vast majority of the latter were derived from progenitors using Vh1-26 in combination with Vk4-74. Antibodies with the germline sequences of these V-regions had no ANA reactivity. It has been shown previously that non-autoreactive B cells can become autoreactive upon acquiring somatic mutations in their Vh and/or Vl regions (6, 10, 13, 20). In contrast to the findings with Vh1-26/Vk4-74 expressing ANAs, the reversion of other Vh and Vk regions of several monoclonal ANAs to the corresponding germline sequences did not abolish ANA reactivity. The maintenance of ANA reactivity upon reversion into germline sequences was observed with 2 of 3 monoclonal ANAs from aging B6 mice, and with 5 of 6 monoclonal ANAs from GVHD mice. Thus, two pathways can be envisaged for the generation of ANA specific antibody forming B cells (Figure 4). In pathway one, they are derived from B cells undergoing somatic hypermutations in response to a foreign antigen (Figure 4, red line); in pathway two, they are derived from B cell progenitors expressing ANA-specific receptors that escape tolerance induction in the bone marrow, possibly by exposure to BCR crosslinking by nuclear antigens (Figure 4, blue line). Indeed, we showed previously that anti-dsDNA reactive B cells escaped tolerance induction by the crosslinking of their BCRs by a T cell independent type 2-antigen (24).

### Restricted Diversity of ANA Specific B Cells

Inspection of the V-region usage of ANA producing hybridomas shows that many ANAs derived from aging mice (21 of 36) (Table 1; Figure 4) and few from mice undergoing a cGVHD (6 of 34) (Table 2; Figure 4) express Vh1-26 in combination with Vk4-74. Interestingly this Vh/Vk combination is expressed in virtually all ANAs from aging mouse 1 (11 of 12), aging mouse 3 (6 of 6), and from the GVHD mouse 3 (5 of 5). Inspection of the V-regions strongly suggests that these ANAs are generated from single B cell clones with the exception of one clone of the GVHD mouse 3, which differs in the CDR3 compared to the other four. In other mice, ANAs are clearly generated from multiple B cell clones. In aging mouse 6, one ANA expresses the Vh1-26/Vk4-74 combination, one expresses the Vh1-26 with Vk4-61, two express the Vk4-74 with Vh1-50 and Vh3-6, respectively, and five express different Vh/Vk combinations. In GVHD mice, all except one mouse (No. 3) express ANAs derived from multiple B cell clones. Thus, in aging B6 mice, the ANA repertoire tends to be restricted, while in GVHD mice, the ANA repertoire tends to be less restricted. The more important question to be answered is why ANAs are oligoclonal in some mice and polyclonal in others. One reason for this restricted repertoire might be that Vh1-26 and Vk4-74 genes are overrepresented in the B cell repertoire of B6 mice in general. RNA sequence analysis of Vh1 usage by B cells from young and aging B6 mice showed that around 10% of these used Vh1-26 (our unpublished results). Since about 50% of all B cells use a Vh1 family member, Vh1-26 is expressed by 5% of them. We did not attempt to verify this number in the hybrids generated, by, for example, cloning and sequencing the Vh regions of random IgM or IgG antibodies not reacting to nuclear antigens. With respect to Vk4-74 usage, Aoki-Ota et al. (31) analyzed the kappa light chain repertoire in B6 mice. This analysis revealed that B6 B cells rarely use the Vk4-74 gene. We also have determined the Vk usage in developing B6 B cells and found that Vk4-74 was used less than 1% in single Vk-rearrangements of preBII cells and immature B cells of the bone marrow [unpublished observation, (32)]. Thus, the restricted ANA-reactive B cell repertoire does not simply appear to reflect a selective usage of the Vh1-26 and the Vk4-74 genes by the B6 B cells. Instead, and more interestingly, this restricted ANA repertoire may occur through selection by and as yet to be defined T cell dependent antigen.

Many years ago, it was shown that haptens such as NP (4-hydoxy-3-nitrophenyl)acetyl (33, 34), oxazolone (2-phenyl-5-oxazolone) (35, 36), and arsonate (p-azophenyl-arsonate) (37, 38) could elicit an oligoclonal humoral immune response, at least in certain inbred strains of mice. Based on this, one might envisage that a hapten-like structure is responsible for the restricted ANA-reactive B cell repertoire in aging B6 mice. Thus, one could imagine that the frequency of ANA-producing B cells that are derived from hapten-induced memory cells in aging mice (red pathway in Figure 4) is higher than the frequency of B cell progenitors with receptors for nuclear antigens that escape tolerance induction in the bone marrow (blue pathway in Figure 4).

### Activation of ANA-Producing B Cells

In ANA-producing mice, B cells expressing nuclear antigen-specific BCRs must be activated by nuclear antigens and helper T cells. In GVHD mice, the alloreactive T cells act as helper T cells. In their initial report on the “allogeneic effect,” Katz et al. reported a drastic enhancement of IgG responses as a result of a GVH reaction (39). Later, Osborne and Katz showed that a simple non-immunogenic hapten–polypeptide conjugate might elicit a vigorous primary IgG response as a consequence of the allogeneic effect (40). Likewise, Hamilton and Miller reported that otherwise tolerogenic hapten-conjugated syngeneic mouse erythrocytes would elicit a strong primary antibody response as a result of a GVH reaction (41). It is possible that this type of general B cell help by alloreactive T cells contributes to the tendency of the ANA response in cGVHD mice to be more diverse.

In aging mice, the specificity and origin of the T helper cells that are required for ANA production is not known. Based on the IgG class of the ANAs produced in these mice, it is conceivable that the helper cells involved in the ANA response are Th1 cells (30).

### Conclusions Regarding the Structure of Anti-Histone Antibodies

Almost 30 years ago, Weigert and coworkers showed that arginine residues in CDR regions play a crucial role for the specificity of anti-dsDNA autoantibodies (10, 11). In the present study, we find an important role of arginine in the Vk-region of certain ANAs. Site-directed mutagenesis revealed that somatic mutations in the Vk4-74 light chains of the Vh1-26/Vk4-74 mAbs determine ANA reactivity, since conversion into their germline sequence completely abolished ANA reactivity. Interestingly, and highly significant, 20 of 23 Vh1-26/Vk4-74-expressing ANAs derived from aging B6 mice had a serine to arginine mutation at position 30 in the CDR1 region of the Vk4-74 gene. In 1 of these 23 mAbs, this change to arginine was the only mutation present in the entire Vk4-74 gene. The finding that one of these (17A9E6) mAb loses ANA-binding activity upon conversion of this arginine into serine strongly indicates the importance of this mutation for ANA reactivity. Furthermore, the introduction of single serine to arginine mutations in the germline configuration of three different mAbs conferred their antinuclear reactivity. This proves that this single mutation can convert an ANA-negative mAb into a positive one and clearly shows how easily Vh1-26/Vk4-74 using mAbs may become autoantibodies. The ANA-reactive B cell repertoire has also been analyzed in B6 mice congenic for a SLE susceptibility locus. Also this analysis revealed a rather frequent usage of Vk4-74 light chains. Thus, Liang et al. (12) found that 9 of 30 ANA producing hybridomas used Vk4-74 light chain (herein called *ai4*). Moreover, it was shown that 3 of these 9 had a serine to arginine mutation at position 30 in the CDR1. In a report by Guo et al. (13), 9 of 33 ANA-producing hybridomas used a Vk4-74 light chain (herein also called *ai4*). At least 5 of these 9 had a serine to arginine mutation at position 30 in the CDR1, like we report herein.

However, since 5 of 6 Vh1-26/Vk4-74 using mAbs derived from the GVHD mice did not have this serine to arginine mutation, other mutations in the Vk4-74 gene can be involved in ANA reactivity.

Yet, another interesting finding reported here is that a high percentage of ANA-reactive mAbs not using the Vh1-26 gene, especially in the repertoire derived from GVHD mice, possess a germline-encoded negatively charged Vh CDR2 region. Thus, 14 of the total 34 monoclonal ANAs (41.2%) derived from the GVHD mice and 6 of 36 (16.7%) derived from the aging B6 mice have such a CDR2 region. Within the total Vh gene repertoire of B6 mice, 11.5% possess such a negatively charged CDR2 (42). Thus, our findings suggest that B cells with such a charged CDR2 might be positively selected into the ANA-reactive B cell repertoire, especially in GVHD mice. The finding that mutations of these negatively charged amino acids into glycine can result in a complete loss of ANA binding supports this hypothesis and, moreover, indicates the potential importance of this region in ANA reactivity. However, additional structural effects of the amino acid change to glycine cannot be excluded, especially since glycine is known to be a helix breaker.

It is noteworthy that despite high ANA serum titers, aging B6 mice do not show the typical lesions that are observed in multiple organs of SLE patients, whereas the GVHD mice do show this. Therefore, one could envisage that the ANA repertoire difference between aging B6 and GVHD mice is playing a role in the pathogenesis of the SLE-like disease observed in GVHD mice.

Overall, the findings described in the present study highlight various new characteristics of ANA-reactive B cell repertoires and argue for similar mechanisms of ANA generation in SLE patients. This might improve our understanding of the pathogenesis of this disease, thereby opening new concepts and therapies for its control.

## Ethics Statement

The State veterinary authorities of Basel (Kantonales Veterinäramt, Basel-Stadt) had approved all animal experiments under permission numbers 1888 and 2434.

## Data Availability

Sequencing data can be found in GenBank under accession numbers MG733774—MG733908.

## Author Contributions

AR conceived the study and designed experiments. All authors performed experiments and analyzed the data. AR, JA, and FK wrote the paper.

## Conflict of Interest Statement

The authors declare that the research was conducted in the absence of any commercial or financial relationships that could be construed as a potential conflict of interest.

## Supplementary Material

Tables S1 and S2 Nucleotide sequences and their translation into amino acids of Vh- and Vk-regions of all hybridomas described herein, which were derived from aging B6 mice.

Tables S3 and S4 Nucleotide sequences and their translation into amino acids of Vh- and Vk-regions of all hybridomas described herein, which were derived from young B6 × bm12 mice undergoing a GvH reaction.

Table S5 Sequences of primers used for cDNA synthesis of total mRNA obtained from ANA-reactive hybridomas. The sequences of primers used for sequencing of Vh- and Vk-regions of the obtained cDNA are also shown.
