# Supplementary material for: Two Distinct Pathways in Mice Generate Antinuclear Antigen-Reactive B Cell Repertoires
Source: Front Immunol. 2018 Jan 22;9:16. doi: 10.3389/fimmu.2018.00016 (PMC5786517; doi:10.3389/fimmu.2018.00016)
Supplement: Table S5 — Sequences of primers used for cDNA synthesis of total mRNA obtained from ANA-reactive hybridomas. The sequences of primers used for sequencing of Vh- and Vk-regions of the obtained cDNA are also shown. [file Table_5.docx]

**Table S5.** Sequences of primers used for cDNA synthesis of total mRNA

obtained from ANA-reactive hybridomas. The sequences of primers used for

sequencing of Vh- and Vk-regions of the obtained cDNA are also shown

| **Primer:** | **Sequence** |
| --- | --- |
| Igκ _C reverse | GATGGTGGGAAGATGGATACAG |
| 5’ L-Vκ3 Fwd | TGCTGCTGCTCTGGGTTCCAG |
| 5’ L-Vκ4 Fwd | ATTWTCAGCTTCCTGCTAATC |
| 5’ L-Vκ5 Fwd | TTTTGCTTTTCTGGATTYCAG |
| 5’ L-Vκ6 Fwd | TCGTGTTKCTSTGGTTGTCTG |
| 5’ L-Vκ6,8,9 Fwd | ATGGAATCACAGRCYCWGGT |
| 5’ L-Vκ14 Fwd | TCTTGTTGCTCTGGTTYCCAG |
| 5’ L-Vκ19 Fwd | CAGTTCCTGGGGCTCTTGTTGTTC |
| 5’ L-Vκ20 Fwd | CTCACTAGCTCTTCTCCTC |
| 5’ L-Vλ1 Fwd | TTGTGACTCAGGAATCTGCA |
| Igλ _C Rev | CTCGGATCCTTCAGAGGAAGGTGGAAACA |
| V_H_ Fwd | GGTSMARCTGCAGSAGTCWGG |
| γ1 C Rev | CTCGGATCCTTCAGAGGAAGGTGGAAACA |
| γ3 C Rev | CTTTGACAAGGCATCCCAGT |
| γ2a C Rev | CCAGGCATCCTAGAGTCACC |
| γ2b C Rev | CCAGGCATCCCAGAGTCACA |
| Murine β-Actin Fwd | GAAGTCTAGAGCAACATAGCAC |
| Murine β-Actin Rev | GTGGGAATTCGTCAGAAGGACTCCTATGTG |
| pJET Rev | ATCGATTTTCCATGGCAGCT |
| T7universal Fwd | TAATACGACTCACTATAGGG |
